# Additional kinetic energy harvesting with extra electrodes by single electrode droplet-based electricity generator (SE-DEG)

**DOI:** 10.1016/j.heliyon.2024.e24765

**Published:** 2024-01-17

**Authors:** Huimin Zhang, Nan Zhang, Zhourui Liu, Ke Jiang, Xiaofeng Zhou

**Affiliations:** a School of Integrated Circuits, East China Normal University, Shanghai, 200241, China; b School of Integrated Circuits, Tsinghua University, Beijing, 100084, China

**Keywords:** Additional kinetic energy, Extra electrodes, Electrical characteristics, Single electrode droplet-based electricity generator

## Abstract

The utilization of water energy through the Single Electrode Droplet-Based Electricity Generator (SE-DEG) represents a universal and high-efficiency method for water energy harvesting. Previous research has extensively elucidated the working principle of SE-DEG based on bulk effect. However, scant attention has been paid to the investigation of the electrical characteristics surrounding the SE-DEG. Remarkably, the electrical characteristics around the SE-DEG can be exploited to generate electricity and harvest corresponding energy. Here we evaluate the electrical characteristics around the SE-DEG by arranging extra electrodes. An interesting phenomenon is found that, on the premise of no contact between extra electrodes and the droplet, there is opposite electricity output from extra electrodes synchronously when the droplet contacts on the PTFE film and SE-DEG electrode and outputs the electricity. This phenomenon is comprehensively explained and verified from working mechanism, the impacts of different arrangements and the array design of extra electrodes. Significantly, utilizing the electrical characteristics could harvest additional kinetic energy with extra electrodes in SE-DEG. This investigation is expected to provide new insights into the future harnessing of water kinetic energy within the SE-DEG framework.

## 1 Introduction

Given the escalating energy crisis and growing concerns about environmental issues, the pursuit of renewable and environmentally friendly energy sources has become an imperative trajectory for fostering sustainable economic development. Especially, water energy, which has huge storage and contains various forms, has attracted worldwide attention [[1], [2], [3], [4], [5], [6], [7], [8], [9]]. Up to now, water energy is able to be harvested by several approaches, for instance, electromagnetic generators [10,11], hydro-voltaic technology [12], electrowetting [13], reverse electrodialysis [14], solid-liquid triboelectric nanogenerators (TENGs) [[15], [16], [17], [18], [19], [20], [21], [22], [23], [24], [25], [26], [27], [28], [29], [30], [31]]. In 2013, the first water-based triboelectric nanogenerator (TENG) was proposed, successfully harvesting water kinetic energy into electric energy [32]. Since then, many researchers gradually explored novel TENGs that can transform water-associated energy into electricity. Droplets, as a kind of widely available form of water, caught the attention of academic circles. In 2014, a prototype droplet-based TENG with superhydrophobic and self-cleaning characteristics was invented and studied [16]. Afterwards, the droplets-driven energy began to be harvested through various approaches. Though these harvesting approaches can effectively convert low-frequency water kinetic energy into electric energy, an inherent interfacial effect in the electricity generation process has been the main bottleneck in limiting their output performance. In 2020, the innovation of droplet-based electricity generator (DEG) based on bulk effect with a transistor-like structure broke the interfacial effect and achieved ultrahigh instantaneous output performance (2.03 kW/m^2^) [33,34]. Later, several DEGs with optimized construction and boosted output performance were proposed and realized more efficient droplet kinetic energy harvesting [[35], [36], [37], [38], [39], [40], [41], [42], [43], [44], [45], [46], [47]]. Single electrode droplet-based electricity generator (SE-DEG) consists of an electrode and a solid surface, which has potential to attain droplet kinetic energy on every solid surface based on bulk effect [48]. Such a universal and simple energy harvester has been explored in our previous work. However, the electrical characteristics around the SE-DEG are unknown and have not been studied systematically.

Here we investigated the electrical characteristics around the SE-DEG by arranging extra electrodes. An electret material of PTFE is selected as the solid surface. On the premise of no contact between extra electrodes and the droplet, there is opposite electricity output from extra electrodes synchronously when the droplet impinges on the PTFE surface, contacts the SE-DEG electrode and outputs the electricity. Through analyzing the working principle of SE-DEG with an extra electrode, we inference that such electricity output from extra electrodes is attributed to electric field change between PTFE and extra electrode under the influence of bulk effect. To evaluate the electric field change, we set three-group experiments to study the relationship between different arrangements of the extra electrode and the electric property (voltage, current and transferred charge). When one extra electrode is located at the *x* = 16 mm, *y* = 20 mm, *z* = 0 mm, the total transferred charge can be elevated up to 26.4 % than the original SE-DEG without an extra electrode. Furthermore, extending one extra electrode into more extra electrodes (such as two extra electrodes) can boost the transferred charge by 50 %, which shows great potential in harvesting additional kinetic energy. We envision that this work provides new insights to enhance water kinetic energy harvesting for the SE-DEG.

## 2 Results and discussion

### 2.1 The structure and performance of SE-DEG with an extra electrode

**Fig. 1:**
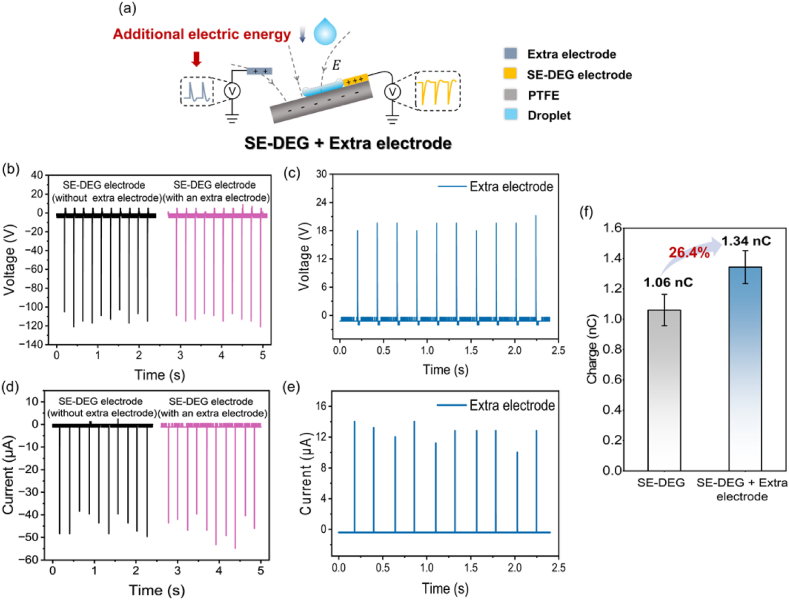
**Design and performance of an extra electrode around the SE-DEG.** (a) The schematic diagram of the SE-DEG with an extra electrode. (b) Time-dependent voltage of the SE-DEG electrode when the SE-DEG works without an extra electrode and with an extra electrode. (c) Time-dependent voltage and current of the extra electrode when the SE-DEG works with an extra electrode. (d) Time-dependent current of the SE-DEG electrode when the SE-DEG works without an extra electrode and with an extra electrode. (e) Time-dependent current of the extra electrode when the SE-DEG works with an extra electrode. (f) Comparison of transferred charge between only the SE-DEG electrode and the SE-DEG with an extra electrode.

The SE-DEG mainly consists of a polytetrafluoroethylene (PTFE) film and an electrode [48]. Being different from our previous work, an extra electrode is arranged surrounding the SE-DEG to explore the electrical characteristics around the SE-DEG (Fig. 1a). Notably, the location of the extra electrode needs to promise no contact between the extra electrode and the droplet. Here, positive voltage is defined as the direction of negative charges along the electric field from the ground to electrode. Positive current is defined as the migration of negative charges from the ground to electrode. When a falling droplet impinges on the PTFE surface and connects the SE-DEG electrode, the droplet and PTFE into a closed-loop system [[33], [34], [35], [36], [37]], the corresponding open-circuit voltage and the short-circuit current of the SE-DEG electrode are measured ∼ −121 V and ∼ −54.8 μA, respectively (Fig. 1b and d, Fig. S1a and 1b). The electric output of the SE-DEG electrode is the result of bulk effect [48]. In the meanwhile, the extra electrode outputs electricity of 21.6 V and 13.7 μA (Fig. 1c and e, Fig. S1c and 1d), opposite to the SE-DEG electrode's output directions. In addition, it is noted that the output of the SE-DEG electrode is almost not influenced by the extra electrode arrangement (Fig. 1b and d). That means arranging the extra electrode is beneficial to enhance energy harvesting of the whole electricity system. We also measured the transferred charge under the condition of with and without an extra electrode. The total transferred charge of SE-DEG with the extra electrode (1.34 nC) is increased by 26.4 % than that of SE-DEG without the extra electrode (1.06 nC) (Fig. 1f). More details will be analyzed from the working mechanism to elucidate the origin of electricity generation from extra electrode.

### 2.2 The working principle of SE-DEG with an extra electrode

**Fig. 2:**
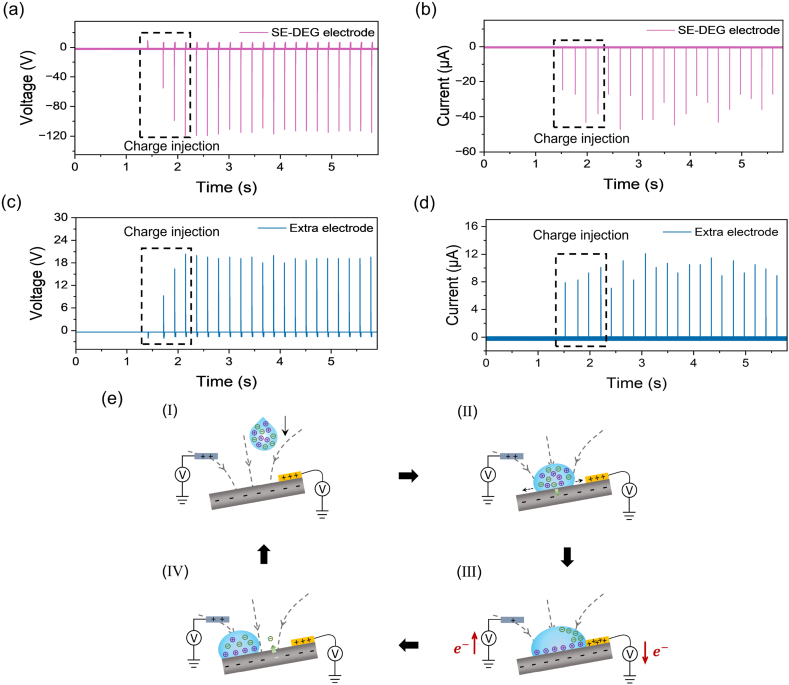
**The working mechanism of SE-DEG with an extra electrode.** (a–b) The voltage and current of the beginning for the SE-DEG electrode. (c–d) The voltage and current of the beginning for the extra electrode around the SE-DEG. (e) The working mechanism of harvesting additional water kinetic energy from an extra electrode around the SE-DEG.

An air-ionization gun is used to neutralize the surface charge of PTFE before the experiment. The initial outputs of the SE-DEG electrode show that first several droplets are released to pre-charge the PTFE surface by triboelectrification (Fig. 2a and b) [34,48,49]. With more droplets falling, the surface charges of PTFE tend to be saturated, resulting in stable outputs of SE-DEG electrode. During the pre-charge process, the PTFE surface is gradually negatively charged as the initial several droplets fall [[49], [50], [51]], which generates a gradually enhanced electric field between the extra electrode and PTFE. Therefore, under the influence of this enhanced electric field, the beginning outputs of the extra electrode are first increasing and then stable (Fig. 2c and d). The charge transfer process in a whole-generation working loop is expatiated in Fig. 2e. Initially, both the SE-DEG electrode and the extra electrode are polarized by the negative charges on the PTFE surface and carry positive charges. Afterwards, the impact of a droplet on PTFE surface results in the formation of an electric double layer (EDL) between the droplet and PTFE surface [[52], [53], [54], [55]]. At this time, the droplet interacts with the surface, which generates triboelectricity [[49], [50], [51], [52]]. However, the whole electricity generation system is open-loop and there is no output to the external (Fig. 2e (Ⅰ) and (Ⅱ)). As soon as the water droplet spreads and contacts the SE-DEG electrode, the droplet connects the SE-DEG electrode and PTFE into a closed-loop [[33], [34], [35], [36], [37]]. Another EDL is formed at the droplet/SE-DEG electrode interface. Positive and negative charges in water directionally move towards the solid surface and SE-DEG electrode, respectively. At this time, a large quantity of negative charges on the PTFE are attracted by positive charges in the droplet due to bulk effect [50,51,[56], [57], [58]]. The potential balance between the ground and the extra electrode is broken. Fewer negative charges on the PTFE can polarize the extra electrode. Therefore, some negative charges migrate from the ground to the extra electrode spontaneously. Accordingly, opposite electricity is generated between the extra electrode and the ground (Fig. 2e (Ⅲ)). With the droplet contracting and leaving the PTFE surface, negative charges at the extra electrode move back to the ground. Some negative charges at the ground move back to the SE-DEG electrode. Partial charges on the PTFE will disappear or be counteracted and the PTFE surface charges will remain stable (Fig. 2e (Ⅳ)). The electricity generation for the next droplet is the same as above process. According to the above analysis, the output of the extra electrode is closely correlated with bulk effect and electric field change between the extra electrode and PTFE. For the SE-DEG without the extra electrode, the thicknesses of dielectric film almost do not affect the output performance [48]. Therefore, as displayed in Figs. S2a and 2b, the open-circuit voltage and short-circuit current of the extra electrode almost remain the same with different thicknesses of PTFE film, meaning that the output is not sensitive to PTFE thickness.

### 2.3 Influence of different arrangements of the extra electrode on output performance

**Fig. 3:**
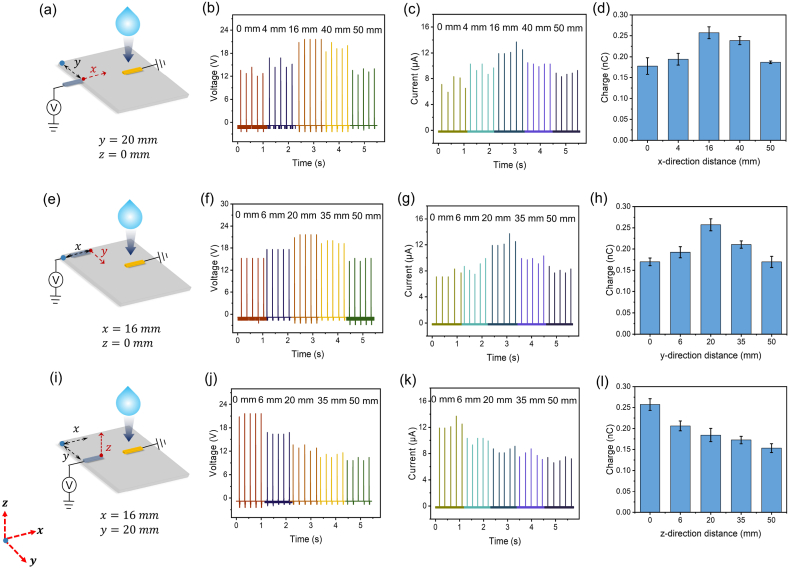
**The electric property of the extra electrode around the SE-DEG vs different arrangement parameters.** (a–d) The relationship of output voltage, current, and transferred charge with various *x*-direction distances (*y* = 20 mm, *z* = 0 mm). (e–h) The electric performance (voltage, current and transferred charge) with various *y*-direction distances (*x* = 16 mm, *z* = 0 mm). (i–l) The corresponding voltage, current and transferred charge under different *z*-direction distances (*x* = 16 mm, *y* = 20 mm).

After elucidating the working principle of the SE-DEG with an extra electrode, the arrangement of one extra electrode on the output performance is further investigated for optimization. The dielectric layer of PTFE is fixed at 100 μm thickness. To demonstrate the arrangement of the SE-DEG electrode and the extra electrode, we take the vertex of the upper left corner of PTFE (the blue point) as the origin and establish a three-dimensional coordinate system. The horizontal direction to the right from the origin is defined as the *x*-direction. Within the PTFE plane, from the vertex of the upper left corner of PTFE, the direction perpendicular to the *x*-direction is the *y*-direction. From the origin, the direction that is vertically upward the PTFE surface is defined as the *z*-direction. In all experiments, the SE-DEG electrode is fixed at *x* = 30 mm, *y* = 30 mm and *z* = 0 mm. The digital pictures of the arrangement of the extra electrode in three directions (*x*-direction, *y*-direction and *z*-direction) are performed in Fig. S3. The setup diagram and variation of output performance under different *x*-direction arrangements of the extra electrode are exhibited in Fig. 3a–d. The extra electrode is set at 5 positions along the *x*-direction. The corresponding voltage, current and transferred charge initially show an increasing trend from 0 mm to 16 mm and then gradually decrease from 16 mm to 50 mm. The optimal arrangement of the extra electrode along the *x*-direction is *x* = 16 mm, which is the closest place to the SE-DEG electrode. The results can be explained that, when the extra electrode gets closer to the SE-DEG electrode where the bulk effect occurs, the electric field between the extra electrode and PTFE is more dramatically influenced by the bulk effect of SE-DEG. More negative charges in the ground are transferred to the extra electrode so that the corresponding outputs of the extra electrode are correspondingly boosted. As the extra electrode moves towards the right across the spreading water drop, the extra electrode gradually moves away from the SE-DEG electrode. The impact of the bulk effect on the electric field established between the extra electrode and PTFE is weakened, leading to the decrease of transferred charges. Next, the variation of the extra electrode along the *y*-direction is also discussed. Fig. 3e performs the diagram in the *y*-direction. The output performance under various values of y (0, 6, 20, 35, 50 mm) is dictated in Fig. 3f–h. The electric performance (voltage, current and charges) is enhanced at first from 0 mm to 20 mm and then gradually weaken with *y* changing from 20 mm to 50 mm. The optimal electric performance is attained corresponding to *y* = 20 mm. Similarly, the reason for such a trend is also attributed to the influence extent of the bulk effect on the electric field between the extra electrode and PTFE. Finally, the output change of the extra electrode moving along the *z*-direction is analyzed. Fig. 3i performs the diagram in the *z*-direction. With *z* varying from 0 mm to 50 mm, all values of electric parameters decrease continuously (Fig. 3j-l). The reason is that the electrostatic induction between the extra electrode and PTFE is weakened with the increase of distance from the extra electrode to PTFE surface, resulting in weaker electric field intensity. At *z* = 0 mm, there is the strongest electric field intensity between the extra electrode and PTFE. To sum up, the optimal extra electrode arrangement is set at *x* = 16 mm, *y* = 20 mm, *z* = 0 mm to realize optimal output. The maximum voltage, current and charges are 21.6 V, 13.7 μA, 0.26 nC, respectively. The total transferred charge of SE-DEG with an extra electrode can be elevated up to 26.4 % more than SE-DEG without the extra electrode. Besides, the working stability of the extra electrode was tested to demonstrate its potential for harvesting additional kinetic energy for a long time. As shown in Fig. S4, the open-circuit voltage can maintain stable output for about 1 h, which shows good stability of the whole electricity generation system.

### 2.4 The output performance of extra electrodes array

**Fig. 4:**
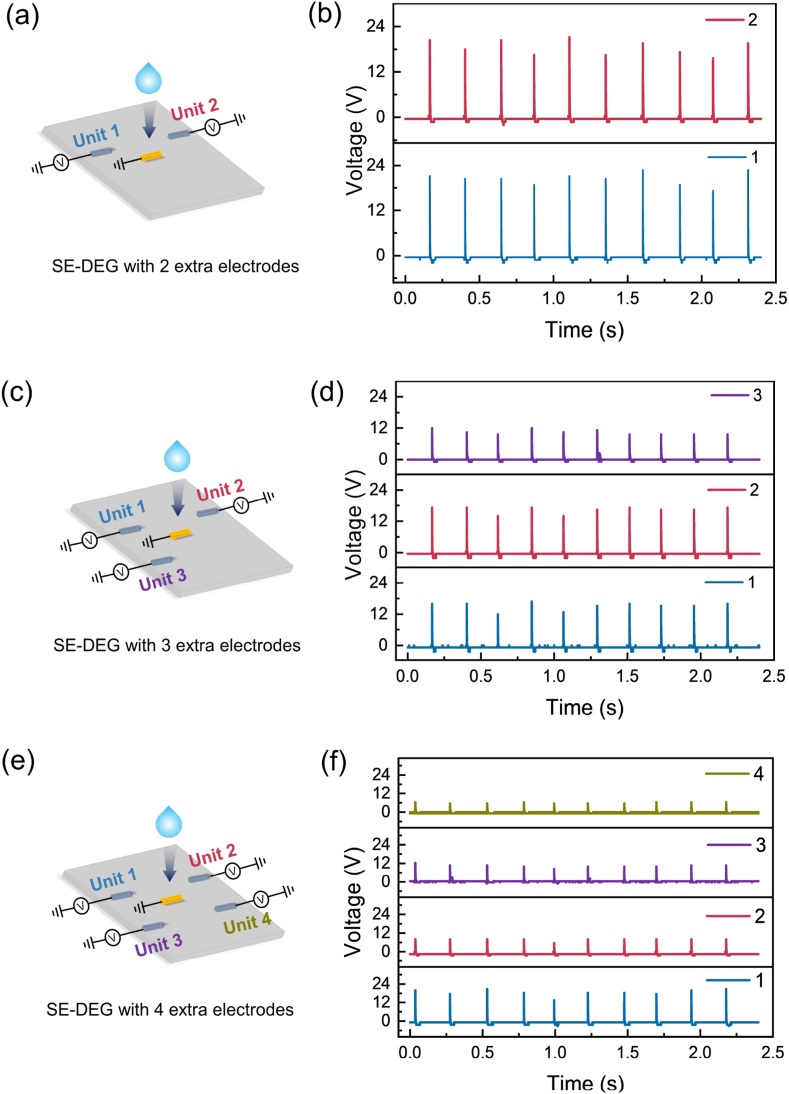
Design and output performance with extra electrodes array. (a–b) Schematic diagram and time-dependent open-circuit voltage with array of 2 extra electrodes. (c–d) Schematic diagram and time-dependent open-circuit voltage with array of 3 extra electrodes. (e–f) Schematic diagram and time-dependent open-circuit voltage with array of 4 extra electrodes.

To harvest the additional kinetic energy as much as possible, the SE-DEG with extra electrodes array is investigated. We first take the array of two extra electrodes as an example (Fig. 4a). The first extra electrode as unit 1 is set at *x* = 16 mm, *y* = 20 mm, *z* = 0 mm. The second extra electrode as unit 2 is fixed at *x* = 40 mm, *y* = 20 mm, *z* = 0 mm. Both units have relatively stable voltage and current output (Fig. 4b and Fig. S5). The voltage and current of unit 1 can reach 21.2 V and 15.2 μA. The maximum voltage and current of unit 2 are 22.8 V and 9.2 μA. Through calculation and analysis, compared with the SE-DEG without an extra electrode, the transferred charge of SE-DEG with two extra electrodes is elevated up to 50 % (from 1.06 nC to 1.59 nC) in Fig. S6. Afterwards, the array of three extra electrodes is also constructed (Fig. 4c). Based on the above SE-DEG with two extra electrodes, for no contact between the droplet and extra electrodes, the third extra electrode as unit 3 is fixed at *x* = 16 mm, *y* = 35 mm, *z* = 0 mm. The voltages of three extra electrodes are shown in Fig. 4d. The output voltages of unit 1, 2, and 3 are distributed in 12 V–17 V. Next, based on the device of SE-DEG with three extra electrodes, the fourth extra electrode as unit 4 is placed at *x* = 40 mm, *y* = 35 mm, *z* = 0 mm (Fig. 4e). The output voltages of these extra electrodes are performed in Fig. 4f. The output voltages of four extra electrodes are 20.8V, 8V, 12.4V and 6.4V, respectively. The slight reduction in the voltage can be explained that some splashes occur when water droplets impinge on PTFE, which has influence on the electricity generation. Moreover, when more extra electrodes are arranged around the SE-DEG, the electric field between every extra electrode and the PTFE may be influenced and coupled to each other. The above results prove that the design of extra electrodes array is beneficial to further harvest additional kinetic energy compared to the design of one extra electrode.

## 3 Conclusion

In this study, we conducted an evaluation of the electrical characteristics surrounding the SE-DEG through the systematic design of various arrangements of additional electrodes. The results clearly demonstrate that by capitalizing on these electrical characteristics, electricity can be generated, and supplementary energy can be harvested through the strategic arrangement of extra electrodes. We explained how the extra electrode generates electricity from the working principle. Also, we analyzed the relationship between different arrangements of the extra electrode and output performance. Finally, the SE-DEG with extra electrodes array was constructed. The design of extra electrodes array further harvests more the additional kinetic energy. We envision that the method of additional kinetic energy harvesting with extra electrodes by SE-DEG will offer valuable new insights into the field of water kinetic energy harvesting.

## 4 Material and methods

### 4.1 Materials

We bought acetone and ethanol of analytical grade from Aladdin and utilized the solutions to start experiments. We purchased some transparent polytetrafluoroethylene (PTFE) films with different thicknesses (100, 300, 1000 μm) of the size of 5 × 5 cm from Yangzhong Fuda Company, China.

### 4.2 Fabrication of device

The PTFE films were first ultrasonically and chemically cleaned using acetone and ethanol for 10 min, respectively. Then an Al wire, as the SE-DEG electrode, was placed on the PTFE surface. The SE-DEG electrode did not contact with the droplet until the spreading area of the droplet reached the maximum. Finally, another Al wire, as the extra electrode, was placed around the PTFE. The extra electrode did not contact the droplet during the whole electricity generation process.

### 4.3 Characterization and electrical measurement

The used water droplets come from the water supply system which was fabricated by a medical infusion pump. In this experiment, the used water was deionized water without specified requirements. The medical infusion pump can adjust the falling velocity of water droplet. The volume of every droplet is 100 μL. In the experiment, we control the droplet at the frequency of 4.5Hz. The falling height of the droplet is fixed at 15 cm. The extra electrode with the aid of a clamp was attached to the 3 axial displacement table to be precisely adjusted from the *x*, *y* and *z* direction, respectively. An oscilloscope (Tektronix, TBS 1102B) with a highly attenuated (100 MΩ) probe was used to assist to show output voltage. The oscilloscope also used to demonstrate the values and waveform of the output current by connecting a low‐noise current preamplifier (Stanford Research System Model SR570). The transferred charge was obtained by the integration of current with respect to the time. During this experiment, the relative humidity (RH) was maintained around 38 % and the environment temperature was set and maintained at 24 °C ± 3 °C.

## Data availability statement

Data will be made available on request.

## Additional information

Additional supporting information may be found online in the Supporting Information section at the end of this article.

## CRediT authorship contribution statement

**Huimin Zhang:** Writing – original draft, Investigation. **Nan Zhang:** Writing – review & editing. **Zhourui Liu:** Validation, Formal analysis. **Ke Jiang:** Writing – review & editing. **Xiaofeng Zhou:** Writing – review & editing, Supervision.

## Declaration of competing interest

The authors declare that they have no known competing financial interests or personal relationships that could have appeared to influence the work reported in this paper.
