# Supplementary material for: Additional kinetic energy harvesting with extra electrodes by single electrode droplet-based electricity generator (SE-DEG)
Source: Heliyon. 2024 Jan 17;10(2):e24765. doi: 10.1016/j.heliyon.2024.e24765 (PMC10831788; doi:10.1016/j.heliyon.2024.e24765)
Supplement: Multimedia component 1 [file mmc1.docx]

**Supplementary information**

**Additional Kinetic Energy Harvesting with Extra Electrodes by Single Electrode Droplet-based**

**Electricity Generator (SE-DEG)**

*Huimin Zhang^1,†^, Nan Zhang^1,†^, Zhourui Liu^1^, Ke Jiang^2^, Xiaofeng Zhou^1,*^*

^1^School of Integrated Circuits, East China Normal University, Shanghai, 200241, China.

^2^ School of Integrated Circuits, Tsinghua University, Beijing, 100084, China.

^†^These authors contributed equally to this work.

**Correspondence**

Xiaofeng Zhou, School of Integrated Circuits, East China Normal University, Shanghai, China.

**Supplementary information**





**Fig. S1.** **Details of output for the extra electrode and the SE-DEG electrode.** (a) Open-circuit voltage of the SE-DEG electrode. (b) Short-circuit current of the SE-DEG electrode. (c) Open-circuit voltage of the extra electrode. (d) Short-circuit current of the extra electrode.





**Fig. S2. The output performances of the extra electrode under different thicknesses PTFE film.** The output voltage and current almost remain the same after using different thicknesses PTFE film, meaning that output is not sensitive to the solid thickness.





**Fig. S3. The digital pictures of the extra electrode around the SE-DEG vs different arrangement parameters.** (a) The arrangement of the extra electrode with different *x*-direction distances (*y* = 20 mm, *z* = 0 mm). (b) The arrangement of the extra electrode with different *y*-direction distances (*x* = 16 mm, *z* = 0 mm). (c) The arrangement of the extra electrode with different *z*-direction distances (*x* = 16 mm, *y* = 20 mm).

**

**

**Fig. S4. Open-circuit voltage under continuous water droplet impingement.**





**Fig. S5. Short-circuit current with array of 2 extra electrodes.**





**Fig. S6. Comparison of transferred charge between only the SE-DEG electrode and the SE-DEG with an extra electrode and SE-DEG with 2 extra electrodes.** It is obvious that whole transferred charges have elevated up 50% than only the SE-DEG as a result of the addition of extra electrodes array.
